# Individualized 3D printing-assisted repair and reconstruction of neoplastic bone defects at irregular bone sites: exploration and practice in the treatment of scapular aneurysmal bone cysts

**DOI:** 10.1186/s12891-021-04859-5

**Published:** 2021-11-25

**Authors:** Guochen Luo, Yao Zhang, Xiahua Wang, Shuaishuai Chen, Dongyi Li, Mingyang Yu

**Affiliations:** 1 Department of Bone and Soft Tissue Oncology, Affiliated Zhongshan Hospital of Dalian University, No. 6 Jiefang street, Dalian, 116001 Liaoning Province China; 2 Dalian Economic and Technological Development Zone, Dalian University, No.10 Xuefu, Dajie, Liaoning China

**Keywords:** Aneurysmal bone cyst (ABC), 3D printing, Bone defect, Irregular bone, Repair and reconstruction, Scapula, Operation planning

## Abstract

**Background:**

The irregular anatomical shape and complex structures of irregular bones make it more difficult to repair and reconstruct bone defects in irregular bones than in the long bones of the extremities. Three-dimensional (3D) printing technology can help to overcome the technical limitations of irregular bone repair by generating simulations that enable structural integration of the lesion area and bone structure of the donor site in all directions and at multiple angles. Thus, personalized and accurate treatment plans for restoring anatomical structure, muscle attachment points, and maximal function can be made. The present study aimed to investigate the ability of 3D printing technology to assist in the repair and reconstruction of scapular aneurysmal ABC defects.

**Methods:**

The study included seven patients with ABCs of the scapula. Based on computed tomography (CT) data for the patient, the scapula (including the defect) and pelvis were reconstructed using Mimics Medical software. The reconstructed scapula model was printed using a 3D printer. Before the operation, the model was used to design the surgical approach and simulate the operation process, to determine the length and radius of the plate and the number and direction of screws, and to determine the bone mass of the ilium and develop reasonable strategies for segmentation and distribution. The operation time, amount of bleeding, length and radius of the plate, and direction and number of screws were recorded.

**Results:**

The average duration of follow-up was 25.6 months, and none of the seven patients experienced recurrence during the follow-up period. The surgical approach, the length and radius of internal fixation, and the number and direction of screws were consistent with the designed operation plan. Patients gradually recovered the anatomical structure of the scapula and function of the shoulder joint.

**Conclusions:**

In the treatment of bone defects caused by irregular bone tumors, 3D printing technology combined with surgery has the advantages of less trauma, short operation time, less bleeding and reducing the difficulty of operation, which can reduce the waste of bone graft, and more complete reconstruction of the anatomical structure of the defective bone.

## Introduction

The term “bone defect” refers to a loss of bone mass due to bone destruction caused by tumors and tumor-related diseases that erode normal bone tissue. Tumors can lead to defects in bones of different sizes, shapes, and irregularities, and irregular bones (e.g., vertebrae, pelvis, jaw, scapula, others) are often located in areas rich in muscle tissue, blood supply, and deep structures. These characteristics make it more difficult to repair and reconstruct bone defects in irregular bones than in the long bones of the extremities.

At present, treatment for bone defects caused by benign bone tumors includes restoration of the integrity and continuity of the defect site via curettage, bone grafting, and internal fixation [1]. However, complete curettage is more difficult and time consuming for irregular bone tumors than for those in the long bones of the extremities. The traditional method of repair and reconstruction takes a long time and is associated with a large amount of bleeding, and the process of autogenous bone grafting is technically demanding. The need to bend the steel plate according to the shape of the defect in different areas further prolongs operation time, increases the amount of blood loss, and increases the risk of surgical infection [2].

In recent years, the rapid development and application of 3D printing technology has greatly improved surgical accuracy, especially in the repair and reconstruction of bone defects. Research has demonstrated that 3D printing technology can overcome the technical limitations of irregular bone repair by generating simulations that enable structural integration of the lesion area and bone structure of the donor site in all directions and at multiple angles [3]. Thus, when repairing and reconstructing bone defects, personalized and accurate treatment plans for restoring anatomical structure, muscle attachment points, and maximal function can be made.

However, despite its widespread application, most studies of 3D printing technology for neoplastic bone defects have focused on the long bones of the extremities, while comparatively few have focused on defects associated with irregular bone tumors. The present study aimed to investigate the clinical effects of 3D printing on the reconstruction of scapular aneurysmal cystic bone defects (ABCs). We hypothesized that the use of such technology would be associated with a good therapeutic effect in terms of the auxiliary repair and reconstruction of irregular bone. Furthermore, we expect our findings to aid in expanding the application of 3D printing technology in the repair and reconstruction of bone defects caused by irregular bone tumors.

## Patients and methods

### Patient data

**Fig. 1:**
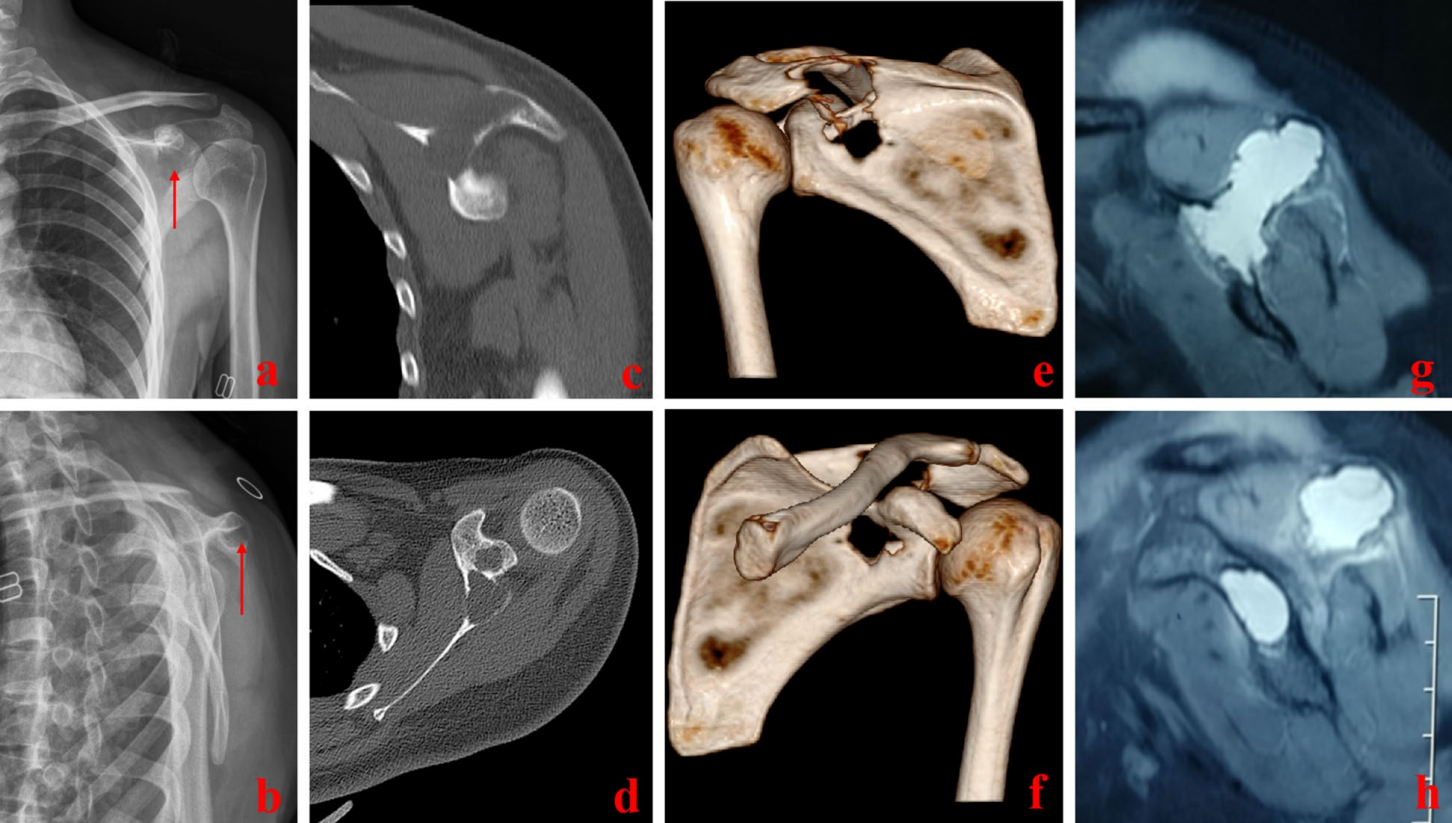
**a** and **b** are DR anteroposterior and lateral radiographs. Low density shadow can be seen in the scapular area with a clear boundary and a sclerosing zone on part of the edge. **c** and **d** show irregular and cystic bone destruction of CT, scapular spine and scapular neck in cross-sectional and sagittal position, respectively, and the bone cortex is punctured before and after perforation. **e** and **f** show three-dimensional reconstruction of the CT scapula, showing severe destruction of the scapula, especially the scapular bone, and penetrating the anterior and posterior bone cortex. **g** and **h** are T2 images of MRI, showing patchy abnormally high signal and lobulated images

The present study included eight patients with scapular ABC treated in our hospital from January 2017 to September 2019. One patient was lost to follow-up, resulting in seven cases finally (three men, four women). Patient age ranged from 18 to 32 years (average: 23.1 years; median: 22 years). When compared with the normal contralateral side, shoulder joint movement was limited on the affected side in all seven patients. Imaging examination revealed that the mass penetrated the scapula and the anterior and posterior cortex of the scapular spine in four cases and the oval shape of the shoulder and back in two patients. Masses were hard, associated with poor mobility, and accompanied by tenderness. The epidermal temperatures of the masses were normal, and there were no obvious abnormalities in the appearance of the skin. All ABCs described in this report were primary lesions, and the possibility of metastasis to the scapula from malignant tumors in other parts or systems was excluded (Fig. 1).

This study was reviewed and approved by the ethics committee of the Affiliated Zhongshan Hospital of Dalian University, and all patients agreed and provided written informed consent.

### Establishment of the model and treatment

#### Model building

Using the patient’s preoperative computed tomography (CT [SOMATOM Definition AS] parameters: kVp,140;mAs,250;slice thickness,0.6 mm;rotation time,0.5;scan time,4.71 s.) data, scapular (including defect areas) and pelvic models were reconstructed for each patient using Mimics Medical modeling software, and the shape, size, and volume of the bone defect were accurately measured. We used the reverse engineering software Geomagic Studio to repair and smoothen the surface of the derived model locally because it was rough. The collected data were compared in terms of parameters such as the shape, size, and volume of the ilium on pelvic 3D CT, following which the corresponding donor site of the iliac bone defect was accurately selected.

#### Printed model and simulated surgery

**Fig. 2:**
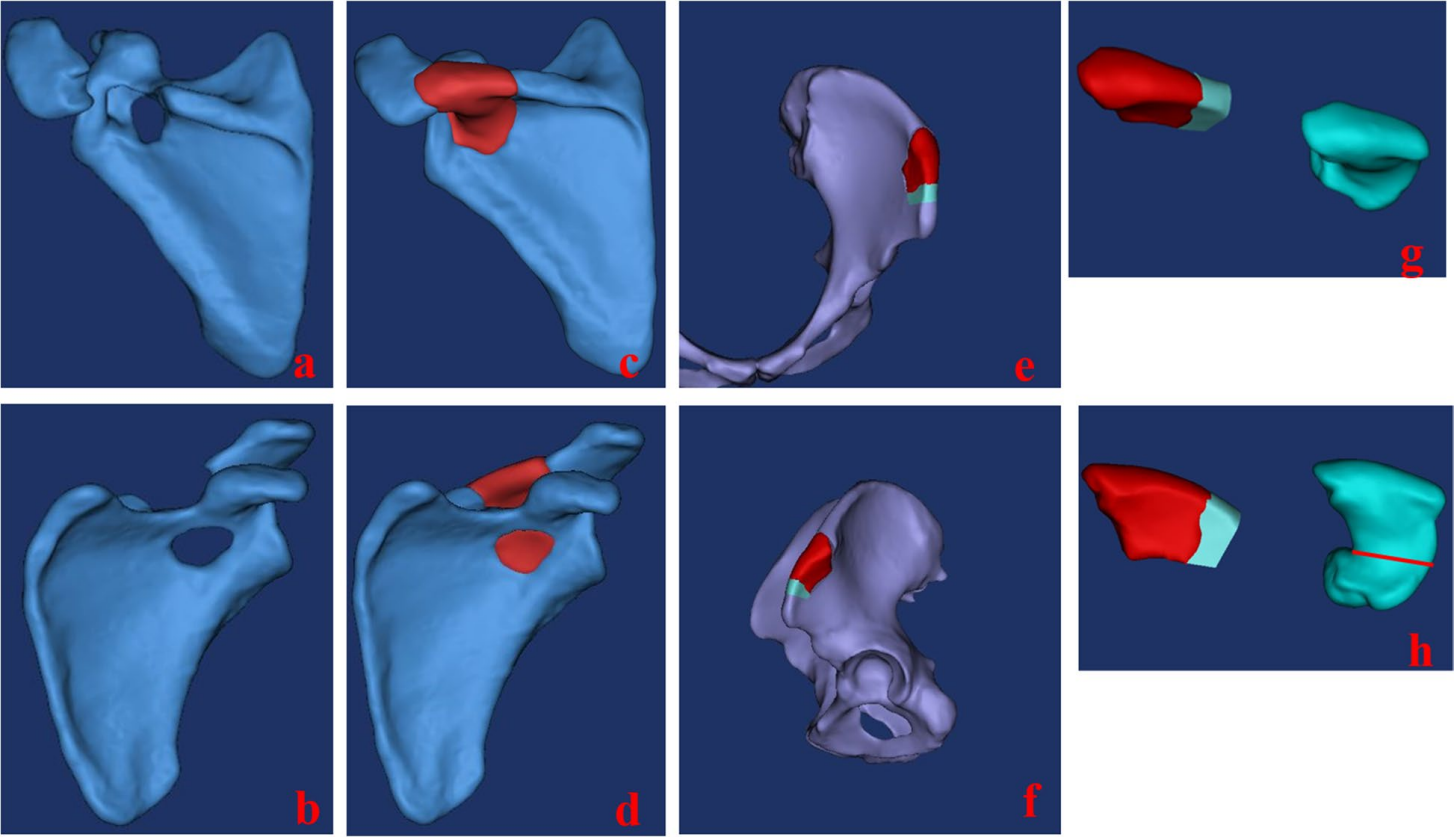
**a** and **b** show the reconstruction model of the affected side of the scapula, and the site of bone destruction can be seen obviously. **c** and **d** show the reconstruction of the defect area of the scapula. **e** and **f** show a simulation of the iliac bone, in which the red part will be used to repair the scapular spine and the green part will be used to fill the gap. **g** and **h** show the defect area and the proposed ilium, the red and green on the left is the ilium to be taken, and the green defect area on the right is reconstructed; the red line in the green part on the right in **h** is the boundary, and the above part is consistent with the shape of the red part of the ilium defect, so as to amputate the ilium for scapula repair

**Fig. 3:**
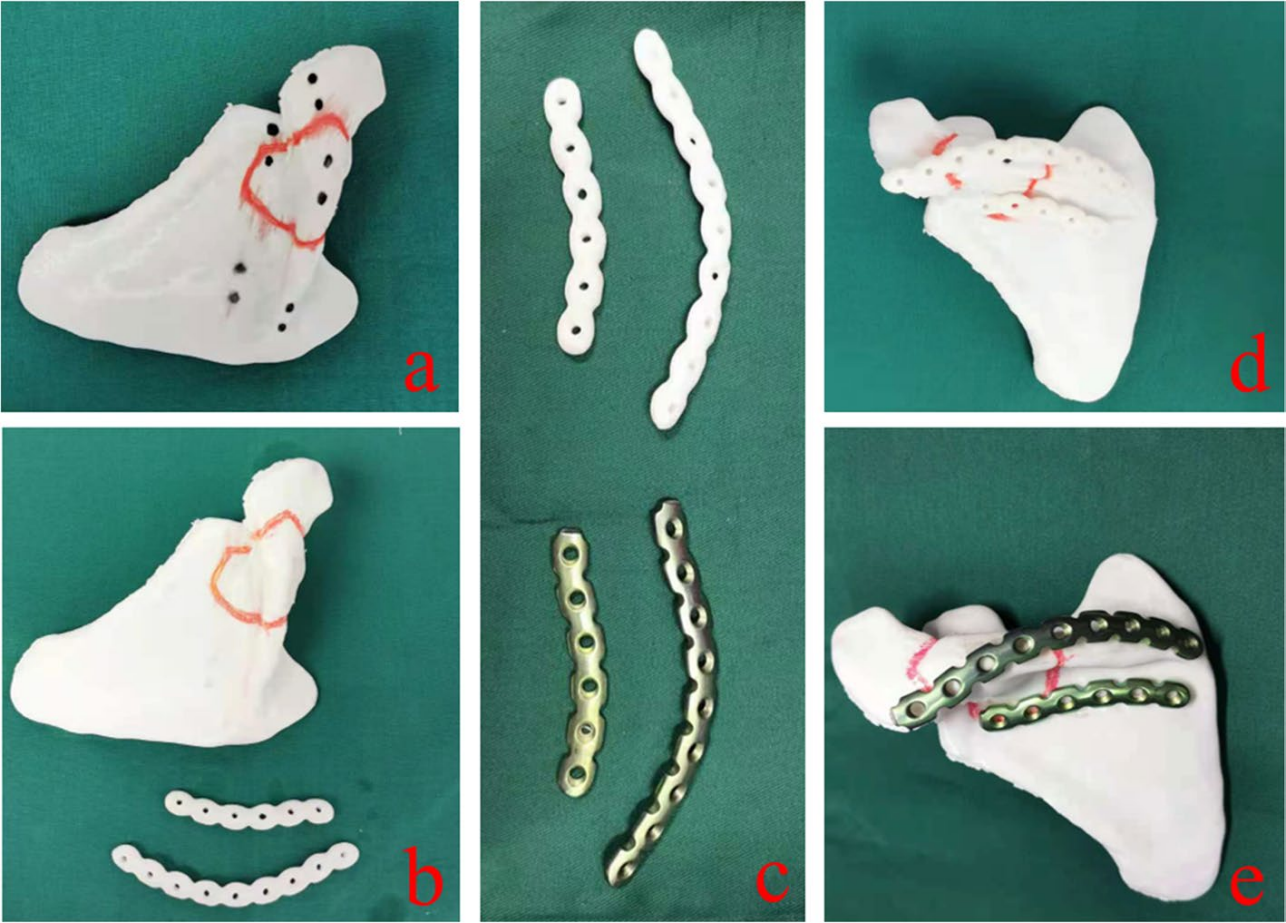
**a** and **b** show the print model, in which the red area is the reconstructed bone destruction area and the black mark is the simulated screw fixation position. **c** shows the prefabricated steel plate according to the model. **d** and **e** are simulated and fixed on the model

After the defect model was reconstructed using Mimics Medical modeling software, the computer-simulated defect repair model and solid defect model were printed and shaped using a 3D printer (Printer, Fused Deposition Modeling [FDM]; material, PLA; print information: layer height, 0.18 mm; first layer height, 0.27 mm; number of outer shells, 2; number of capped layers, 3; number of bottom seals, 3; filling density, 5%; filling shape, hexagonal; combined filling, off; printing speed, 100 mm/s; empty speed, 120 mm/s. Rigid plate model information: X: 86.63 mm, Y: 59.99 mm, Zrig 38.75 mm; print time, 67 min. Model information of scapula: XRV: 103.54 mm, Magi: 127.42 mm, ZRV: 116.37 mm; print time, 251 min.) at a ratio of 1:1. A surgical plan was then developed based on the 3D model, pathological results, and imaging data. The location, volume, and shape of the defect were accurately evaluated, following which the length, radius, screw number, and direction of the surgical approach and internal fixation plate were determined. After printing the plate model, the operation was simulated on the 3D-printed solid model, and the individual anatomical plate was customized. The surgeon focused on the internal fixation position and direction of the steel plates and screws to determine the optimal position and direction of fixation. Practice was maintained until the day of the operation (Figs. 2, 3).

#### Operation

**Fig. 4:**
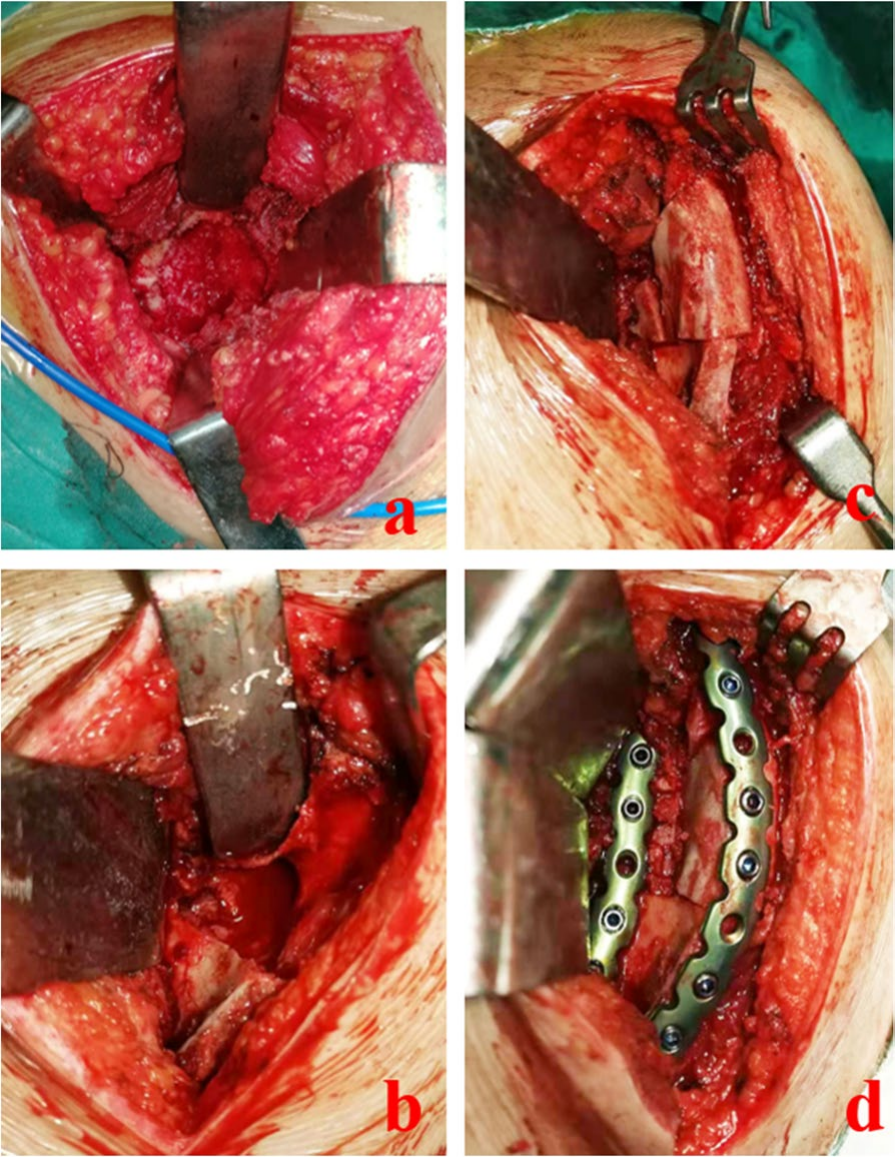
**a** shows the lesion that was exposed intraoperatively, **b** shows the lesion after curettage, **c** shows the iliac bone matching the scapular defect area, and **d** shows the plate and screw fixation

Pathological findings revealed that all scapular masses were ABCs in all seven patients. According to the preoperative plan, an iliac bone equivalent to the size, shape, and/or volume of the defect model was obtained and chiseled for use. At the same time, curettage of the scapular mass was performed, and bone defects of the scapular and scapular body were observed during the operation. In some patients, a floating acromion in which the defect was filled with fibrous tissue or blood clots was observed, along with pulsatile bleeding or vascular tissue. The diseased tissue was removed, and the diseased tissue in the defective area was completely scraped from the normal cancellous bone surface. The iliac bone was trimmed and polished to match the shape of the two defect surfaces. After ensuring traction of the upper limb, the fractured pieces of the scapula and scapular body were placed and tamped. After confirming a good match between the bone and scapula, the bone was polished with a file to prevent friction against the muscles and fixed with plates and screws. Four cases of severe bone destruction were treated via curettage and bone grafting (mixed autograft and allograft) of the scapular ABC, followed by reconstruction and internal fixation of the scapula plus iliac osteotomy. In addition, three patients with small defect volumes underwent curettage of the scapular ABC, bone grafting, reconstruction, and internal fixation of the scapula plus iliac osteotomy (Fig. 4).

### Rehabilitation training and evaluation of curative effect

Gradual rehabilitation training began 3 days postoperatively. After discharge, patients underwent follow-up, evaluations of shoulder function, and rehabilitation exercise guidance at outpatient revisits.

**Fig. 5:**
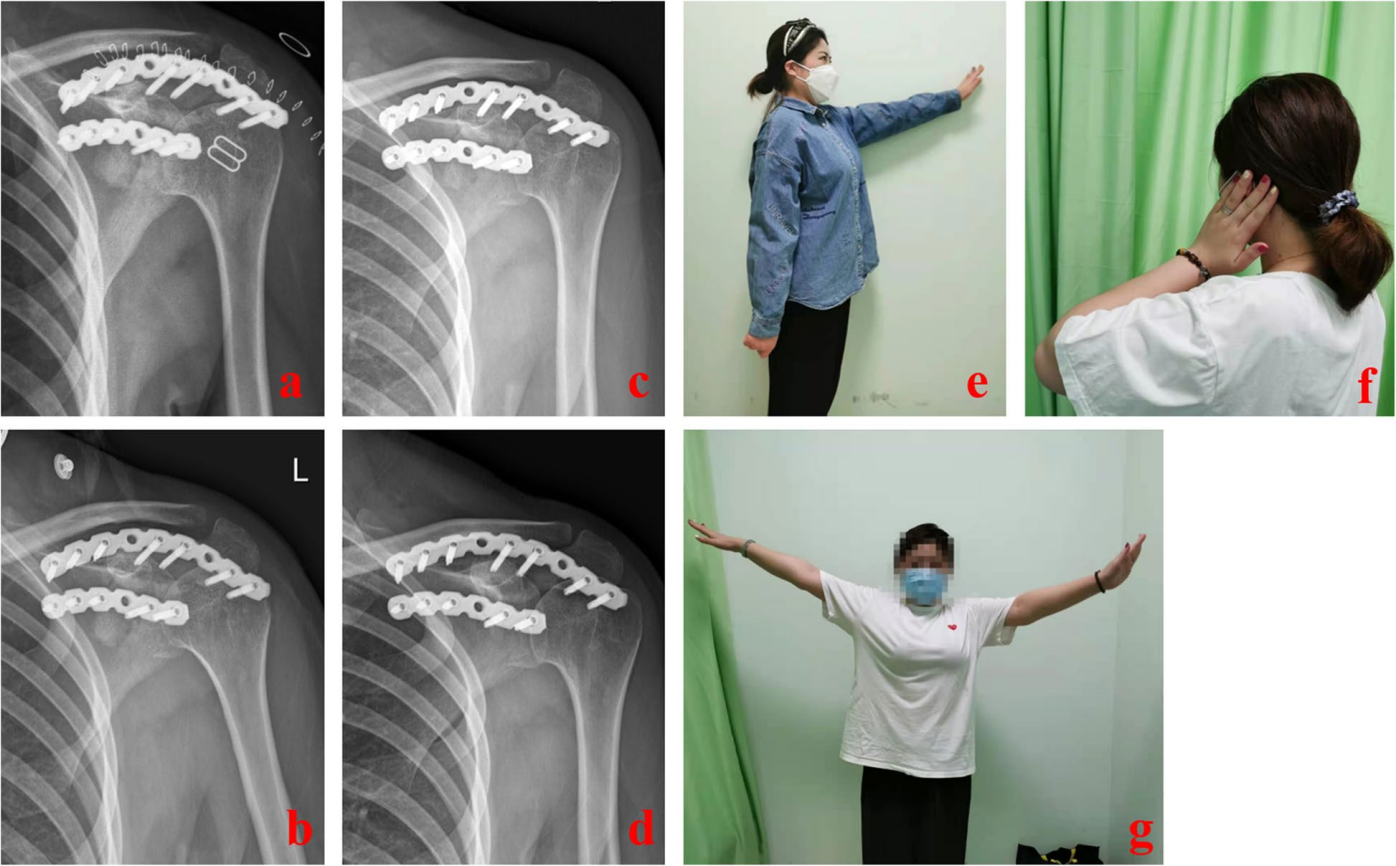
**a**-**d** are DR films reexamined at 1 week, 1 month, 3 months and 12 months after operation, respectively. It can be seen that the density of the bone graft area of defect repair and reconstruction decreases gradually, and at 12 months, the density is the same as that of the surrounding normal cancellous bone. There is no obvious boundary between the tumor cavity and the surrounding normal bone tissue, and the tumor cavity basically disappears. **e**-**g** show the functional recovery of the patients 1 year after operation, showing the gradual recovery of shoulder abduction, external rotation and protrusion function of the patients

Based on imaging evaluations performed 1 year after surgery (tumor cavity boundary, bone graft fusion, and absorption) and Constant–Murley shoulder joint function scores (including pain, daily activities, active range of motion, and muscle strength), treatment effects were divided into three levels: excellent, good, and poor [4]. Excellent effects were defined as follows: ability to resume work, disappearance of the tumor cavity boundary, good bone graft resorption, Constant–Murley score ≥ 80 points. Good effects were defined as follows: ability to return to general life and work, blurred tumor cavity boundary, Constant–Murley score of 60–79 points. Poor effects were defined as follows: inability to return to work, clear tumor cavity boundary, poor bone graft resorption, Constant–Murley score < 60 points (Fig. 5, Table 1).

**Table 1:** Clinical data of 7 patients

Patient	Gender	Age (yr)	Size (cm)	Grafts	Surgical time (min)	Intraoperative blood loss (ml)	Follow up (month)	Score^a^
1	Female	32	4*1.2*4.3	Autoglaft, Allograft	125	240	12	82
2	Female	22	3.1*0.9*2	Autoglaft	96	185	31	81
3	Male	18	3*1.2*1.3	Autoglaft	106	200	23	78
4	Male	27	4*0.5*3.4	Autoglaft, Allograft	115	210	18	82
5	Female	19	3.4*2.5*2.5	Autoglaft, Allograft	100	195	20	84
6	Male	24	3.8*1.2*3	Autoglaft, Allograft	110	205	33	90
7	Female	20	2.7*0.8*1.9	Autoglaft	85	165	42	88

^a^The total score was 100, of which the imaging evaluation accounted for 20% of the total score, and the shoulder function score of Constant-Murley accounted for 80% of the total score.

## Results

After discharge from the hospital, follow-up was conducted in an outpatient clinic. The follow-up time ranged from 12 to 42 months, and the average follow-up time was 25.6 months. No local recurrence or distant metastasis was observed in any of the patients until the last follow-up. Scapular lesions exhibited a good appearance, and the plates and screws were well fixed. There were no complications such as infection, internal fixation slippage, broken nails, or broken plates. Implanted iliac bone and/or allogeneic bone was gradually absorbed, and the function of the shoulder joint gradually recovered. The statistical data were processed by SPSS23.0 statistical software. Among all seven patients, the operation time was 120 ± 18 min, while the level of intraoperative blood loss was 270 ± 40 ml. The incision approach was consistent with the preoperative plan, and the internal fixation materials used were designed prior to the operation according to the 3D-printed model. The range and volume of iliac bone removal and the number, position, and curvature of implants (such as plates and screws) were consistent with the preoperative plan. Six cases exhibited excellent outcomes, while one exhibited a good outcome.

## Discussion

Based on the patient’s CT or magnetic resonance imaging (MRI) data, 3D printing technology (also known as rapid prototyping [RP]) can be used to manufacture physical models using a layer-by-layer approach. The data are first processed through computer software to construct a 3D model, following which a 3D printer is used to generate the physical model [5, 6]. Recent advancements in computer science and printing technology have increased focus on the application of 3D printing to orthopedics, especially for the repair and reconstruction of bone defects [7]. Bone tumor specialists now combine their knowledge of 3D printing, treatment of traumatic orthopedic fractures, and repair and reconstruction of tumor-related bone defects to perform comprehensive and multi-angle evaluations based on 3D images and printed physical models. Such models can then be used to develop precise and individualized treatment plans; design surgical approaches; determine the length, arc, number, and direction of screws; determine the amount of iliac bone removal; perform reasonable segmentation and allocation; improve the safety and accuracy of operation; reduce operation time, trauma, and bleeding; and reduce the risk associated with operation [5]. Application of 3D printing technology to clinical orthopedic treatment thus provides a personalized and accurate surgical scheme for managing difficult and complex orthopedic diseases, especially when patients require repair and reconstruction of bone defects [8]. The present results demonstrate that 3D printing technology combined with surgery has the advantages of less trauma, short operation time, less bleeding and reducing the difficulty of operation, which can reduce the waste of bone graft, and more complete reconstruction of the anatomical structure of the defective bone.

The complex and varied morphological structure of irregular bone is associated with unique circumstances when attempting repair and reconstruction of tumor-related bone defects. Irregular bone provides the starting and ending point of many important muscular tissues, and such bone is surrounded by important nerves, blood vessels, and other tissue structures. Most irregular bones are located in important functional areas, including those involved in organ protection, movement, and weight-bearing functions. Therefore, when an irregular bone is injured or defective, its special anatomical structure increases the difficulty of treatment. Failure to restore its morphological structure will lead to the loss of some important functions and substantially reduce quality of life in affected patients [9]. ABCs are benign bone tumor-like lesions with unknown etiology and are highly invasive and destructive [10]. First reported by Jaffe et al. [11] in 1942, ABCs account for approximately 1% of bone tumors, which are mainly concentrated in the long bones of the extremities and spine, while cases occurring in irregular bones such as the scapula are rare [10, 12]. Although ABCs are benign, they often exhibit invasive behavior and a tendency to relapse. These invasive and recurrent characteristics often lead to more serious bone destruction, and severe cases can be associated with pathological fractures, which can further complicate treatment [13].

Advancements in 3D printing technology combined with innovative digital imaging equipment have greatly promoted the treatment of tumor-related defects in irregular bone: Specialized software can be used to observe the locations of peripheral nerves and blood vessels in various directions and angles, helping to avoid accidental injury to these structures during the operation and allowing for accurate measurement of the volumes of the tumor and bone defect. Not only does this provide a basis for clarifying the focus prior to surgery based on the printed model, but it also helps to determine the length and radius of the steel plate as well as the number and direction of screws, thereby allowing for a customized and accurate operation plan [14, 15]. In addition, the operation can be simulated according to the operative plan, allowing for constant improvements that can help to determine the optimal incision position and direction/position of internal fixation. These characteristics can in turn reduce operation time, blood loss, and the risks associated with surgery and compounded medical expenses. Furthermore, 3D printing technology can reduce the risk associated with surgical operation: The preoperative chief surgeon can use the model to communicate and explain the entire operation process and its important nodes to the team, including anesthesiologists, instrument nurses, and internal fixation suppliers. Use of the model during simulation can help the entire surgical team improve their surgical skills and efficiency before the operation. During the operation, the model can be placed on the operating table after disinfection, allowing for reference at any time during the operation. Previous studies have also reported that the adhesion of the pre-bending internal fixation is better using printed models, and that the fixation is firm and not easily loosened. This provides a stable local mechanical basis for bone regeneration and reduces peeling and injury of soft tissue, operation and intraoperative bleeding times, the duration of anesthesia, the frequency of C-arm arm, and the dosage of antibiotics during operation [15–17]. Tserovski et al. [5] used 3D printing technology combined with printed models to assist in the diagnosis and treatment of hip revision. The authors reported that this not only improved the accuracy of diagnosis but also helped the team to determine the type and size of the prosthesis before operation.

In addition, 3D printing technology can aid in matching the donor site ilium. That is, the defect site can be matched with the iliac bone using the computer, and a scapular bone defect site with the same shape or volume as the iliac bone extraction site can be accurately selected. The body surface can then be marked before the operation for faster and more accurate extraction, reducing the operation time and the extent of surgical trauma [18, 19]. Lastly, 3D printing can help to improve the efficiency of doctor-patient communication: In the past, preoperative communication between doctors and patients was mostly based on the patients’ own X-ray, CT, and other imaging data. Thus, patients and their families without medical knowledge may have difficulty fully understanding their condition and surgical plan [6]. The 3D simulation map and printed model can be used to visually present the structure of the real lesion, allowing patients and their families to gain a more intuitive understanding of the lesion characteristics and treatment plan. Such an understanding will in turn improve doctor–patient communication and the ability of patients to cooperate during the later stages of rehabilitation treatment [15, 20].

In the present study, we only used the 3D printed prosthesis for simulated surgery and for the selection of suitable iliac bones for repair and reconstruction, instead of directly using the 3D printed prosthesis for treatment, mainly for the following reasons: (1) The average age of the patients was relatively young, and the oldest patient was only 32 years old. Although obtaining iliac bone is uncomfortable for the patient, long-term fixation of a foreign body such as a 3D-printed prosthesis can increase both the physiological and psychological burden among patients. Moreover, (2) although the 3D-printed prosthesis more closely approximates the defect than the iliac bone in the early stage and it is easier to fix the muscle and other tissue structures, the iliac bone can be more in line with the physiological structure of the human body after long-term plastic transformation, leading to firmer muscle attachment. In addition, (3) rejection and periprosthetic fractures are inevitable adverse events associated with the use of such a prosthesis. If such events are serious, a second operation may be required, thereby aggravating trauma and increasing the economic burden among patients [21, 22]. (4) Other studies have also indicated that the use of 3D printed prostheses for repair and reconstruction can lead to loosening or displacement of the screws due to long-term activity at the shoulder joint, resulting in insecure fixation of the joints, loosening of the prosthesis, and muscle wear. These events can in turn lead to complications such as chronic pain, which are not conducive to postoperative recovery training [23]. Wang et al. [24] used a computer-assisted 3D-printed hemipelvis prosthesis to treat 11 patients with malignant bone tumors around the acetabulum, two of whom experienced dislocation of the hip prosthesis after surgery. Liang et al. [25] used a 3D-printed pelvic prosthesis to reconstruct bone affected by pelvic tumors after resection. Among the 35 patients in their study, seven exhibited delayed wound healing, while two experienced hip dislocation. In contrast, there is no rejection after repair and reconstruction with autogenous iliac bone, after which the grafted bone can be completely integrated with the scapular bone to achieve bony healing. Furthermore, in autogenous cases, the iliac and scapular bones have the same contact area, and the muscle attaches more firmly to the bone surface. At the same time, complications or adverse reactions such as chronic pain, rejection, and prosthesis loosening can be avoided to the maximum extent, allowing for improved quality of life among patients [26].

Despite our hope that the cases and treatments reported herein can provide a reference for the repair and reconstruction of tumor-related bone defects in irregular bone sites, the present study had some limitations. First, the duration of follow-up was short, and there were no comparisons with other treatment methods. Second, due to the small sample size, no statistical analyses were performed. Finally, there were still differences in Constant–Murley scores due to individual factors such as patients’ sensitivity to pain and the extent to which rehabilitation training had been completed. In future studies, we aim to increase the number of samples, control the range of bone grafts, prolong the follow-up time, explore a more objective evaluation system, and carry out statistical analysis.

## Conclusion

Our results support the notion that 3D printing technology can be applied in the assist repair and reconstruction of bone defects caused by irregular bone tumors. Specific 3D physical models can be printed based on imaging data obtained prior to the operation, combination of anatomy and MRI imaging, enabling surgeons to accurately assess the operative situation and the distribution of blood vessels around the diseased bone. In addition, such technology can be used to select the appropriate personalized internal fixation, to select the appropriate methods for approach and reduction, and to formulate the optimal surgical plan. These characteristics effectively reduce injury to muscles, blood vessels, and nerves during the operation; reduce the difficulty of the operation; shorten the operation time; and reduce bleeding during the operation. Additional studies have indicated that 3D printing technology can improve the safety of the operation by reducing damage to the donor site and decreasing the amount of waste associated with the transplanted bone, thereby reducing the incidence of postoperative complications [2, 15, 20]. Further research will continue to utilize the advantages of 3D-printing technology for the treatment of tumor-related bone diseases in clinical practice.

## Data Availability

All data generated or analysed during this study are included in this published article.

## Abbreviations

3D: Three dimensional
ABC: Aneurysmal cystic bone defect
CT: Computed tomography
FDM: Fused Deposition Modeling
MRI: Magnetic resonance imaging
RP: Rapid prototyping
